# Progesterone depletion results in Lamin B1 loss and induction of cell death in mouse trophoblast giant cells

**DOI:** 10.1371/journal.pone.0254674

**Published:** 2021-07-14

**Authors:** Hiromu Morimoto, Misuzu Ueno, Hideyuki Tanabe, Tomohiro Kono, Hidehiko Ogawa

**Affiliations:** 1 Department of Bioscience, Tokyo University of Agriculture, Tokyo, Japan; 2 Department of Evolutionary Studies of Biosystems Science, School of Advanced Sciences, The Graduate University for Advanced Studies, SOKENDAI, Shonan Village, Hayama, Kanagawa, Japan; University of Massachusetts Amherst, UNITED STATES

## Abstract

Trophoblast giant cells (TGCs), a mouse trophoblast subtype, have large amounts of cytoplasm and high ploidy levels via endocycles. The diverse functions and gene expression profiles of TGCs have been studied well, but their nuclear structures remain unknown. In this study, we focus on Lamin B1, a nuclear lamina, and clarify its expression dynamics, regulation and roles in TGC functions. TGCs that differentiated from trophoblast stem cells were used. From days 0 to 9 after differentiation, the number of TGCs gradually increased, but the amount of LMNB1 peaked at day 3 and then slightly decreased. An immunostaining experiment showed that LMNB1-depleted TGCs increased after day 6 of differentiation. These LMNB1-depleted TGCs diffused peripheral localization of the heterochromatin marker H3K9me2 in the nuclei. However, LMINB1-knock down was not affected TGCs specific gene expression. We found that the death of TGCs also increased after day 6 of differentiation. Moreover, Lamin B1 loss and the cell death in TGCs were protected by 10^−6^ M progesterone. Our results conclude that progesterone protects against Lamin B1 loss and prolongs the life and function of TGCs.

## Introduction

The placenta is essential for sustaining fetal growth during pregnancy, and defects in its function result in fetal growth retardation and fetal death [1]. In mice, the polar trophectoderm differentiates into the subtypes of placenta, including spongiotrophoblast, glycogen trophoblast cells, several labyrinth trophoblast cell types, and trophoblast giant cells (TGCs) [2]. TGCs have paracrine and endocrine effects for both maintenance of the feto-maternal interface and regulation of maternal adaptations to pregnancy [3].

Trophoblast stem (TS) cells are derived from the polar trophectoderm cells of murine blastocysts. TS cells are differentiated into all types of trophoblasts, including spongiotrophoblast cells, labyrinth cells, and TGCs. TGCs are among the terminally differentiated cells in rodent placenta, and they are important for embryo implantation and the promotion of maternal adaptations to pregnancy [3]. TGCs form via the endoreduplication that occurs when a cell undergoes multiple S-phases without entering mitosis and undergoing cytokinesis [4], and endoreduplication results in large polyploidy cells [5]. Examples of endoreduplication are found among protozoa, arthropods, mollusks, and plants. A possible advantage of polyploidy cells is that they have been proposed to act as a tissue envelope, more metabolically active than diploid cells and that they protect against mutations and apoptosis [4]. Murine TGCs accumulate DNA up to 1000C [6]. Recently, DNA content in TGCs showed that small regions of the genome are relatively under-replicated (~5% of the genome) while others are over-replicated (~0.2% of the genome). Interestingly, over-replicated regions are the loci of TGC-specific expressed genes, which are prolactin, serpins, and cathepsins on chromosome 13 [7]. These results suggest that the nuclear structure of TGCs is unique, but the structure itself has remained unclear.

The nuclear lamina is a meshwork of proteins located at the inner nuclear membrane [8,9]. The major constituents of the nuclear lamina are the type-V intermediate filament proteins known as lamins, which include Lamin A/C, Lamin B1, and Lamin B2 [10]. The nuclear lamina is essential for transcription, DNA replication, DNA repair, and chromatin organization [11]. Lamin B plays roles in DNA replication, the formation of the mitotic spindle, chromatin organization, and the regulation of gene expression [12]. In the colon cancer cell line DLD-1, silenced *LMNB1* reduced the localization of the heterochromatin marker H3K27me3 in the periphery of the nucleus [8]. Furthermore, Lamin B1 loss is observed in cellular senescence [13–16] or cell death [17–19], and in some cells LMNB1 depletion induced apoptosis [20,21]. These results indicate that LMNB1 is crucial for cell survival. However, the role of Lamin B1 in polyploidy cells has remained to be elucidated.

In mouse placenta, the number of TGCs increased from days 10 to 14 days post coitum (dpc) and then decreased at day 16 dpc [22]. Detmar et al. [23] showed that TUNEL-positive TGCs increased after 15.5 dpc. These results indicated that the cell death of TGCs started around 16 dpc. TGCs sometimes undergo fragmentation that shows signs of apoptosis [24]. In some reports, TGCs escaped apoptosis that would have resulted from incomplete DNA synthesis or DNA damage by p21/Cip2, which repressed CHK1 [4,25]. Therefore, TGC death might be another type of apoptosis that is related to the alteration of the nuclear structure.

In this study, we examined the role of Lamin B1 on the alteration of the nuclear structure toward cell death in order to clarify the mechanism of cell death in TGCs.

## Materials and methods

### Animal housing

All mice were maintained and used in accordance with the Guidelines for the Care and Use of Laboratory Animals, as specified by the Japanese Association for Laboratory Animal Science and by the Tokyo University of Agriculture (approval number: 2019054).

### Cell culture

Mouse trophoblast stem (TS) cells were derived from a blastocyst (C57BL/6N×DBA/2N) and cultured in TS medium: RPMI 1640 (Nacalai Tesque, Inc., Kyoto, Japan) supplemented with 20% fetal bovine serum (FBS), 1 mM sodium pyruvate (Gibco Invitrogen, Carlsbad, CA, USA), 100 μM β-mercaptoethanol (Sigma-Aldrich, St. Louis, MO, USA), 2 mM L-glutamine (Gibco), 50 U/ml penicillin (Gibco), and 50 μg/ml streptomycin (Gibco) as described previously [26,27]. TS cells were maintained in an undifferentiated state in 70% mouse embryonic fibroblast-conditioned medium (MEF-CM), 30% TS medium containing 25 ng/ml FGF4 (PeproTech EC, London, UK), and 1 mg/ml heparin (Sigma-Aldrich). Differentiation of TS cells was induced by withdrawing FGF4, heparin, and MEF-CM. For steroid hormone treatment experiments, progesterone was prepared as a 10^−1^ M solution in ethanol and added to cells 72 h after withdrawal of FGF4 at final concentrations of 10^−6^, 10^−7^, and 10^−8^ M. Steroid hormones were removed from FBS, which was stripped by charcoal−dextran treatment.

### siRNA transfection

siRNA against *Lmnb1* was transfected with Lipofectamine 2000 (Life Technologies, Carlsbad, CA, USA) reagent according to the manufacturer’s protocol at a final concentration of 10^−7^ M. The siRNA sequences were as follows: siLmnb1 Sense: 5’ -CAGAUGAAACUUUACUUAAAGUGGA -3’ siLmnb1 Antisense: 5’ -UCCACUUUAAGUAAAGUUUCAUCUGCU -3’ Scramble Sense: 5’ CGUUAAUCGCGUAUAAUACGCGUAT -3’ Scramble Antisense: 5’—CAGCAAUUAGCGCAUAUUAUGCGCAUA -3’. siRNA-transfected TS cells were cultured for 72 h in TS medium without FGF4. siRNAs were purchased from Integrated DNA Technologies (Tokyo, Japan).

### Gene expression analysis

Total RNAs were isolated from cells by using ISOGEN (Nippon Gene, Tokyo, Japan) and were treated with DNase (Promega, Madison, WI, USA) to eliminate genomic DNA. cDNA was synthesized from 1 μg DNase-treated total RNA by using Super Script Ⅲ Reverse Transcriptase (Life Technologies). Quantitative gene expression analysis was performed with gene-specific primers using Power SYBR Green Master Mix (Applied Biosystems, Carlsbad, CA, USA) on a QuantStudio 3 system (Applied Biosystems). The expression levels of target mRNAs were calculated from a standard curve and were normalized relative to the amount of *Gapdh*. The primers are listed in Table 1.

**Table 1:** Primers used for quantitative gene expression analysis.

	*Primer sequences*			
*Genes*	*Forward*	*Reverse*	*Annealing temp(°C)*	*Size(bp)*
*Cdx2*	GGAAGCCAAGTGAAAACCAGGA	TGGCAGCCAGCTCACTTTTC	60	133
*Ascl2*	TTAAGGGCTGAGCACCAGGA	CCAGTCAAGGTGTGCTTCCAT	60	186
*Tpbpa*	CTGAACTGCAAGAGCAGAAGGATA	AACTGGCTGTGGTTTGTTTTCC	60	224
*Gcm1*	CAACTGCAATGGACCCCTGA	CATGCTCGCCTTTGGACTGG	60	116
*Syna*	GAGCTCGTGAACCATAACCGA	TGTGGTTTGGGGGAAACTACC	60	144
*Prl3d1*	TACCCTGCTTGGTCTGGACT	GGGCACTCAACATTCGTTCT	65	167
*Prl2c2*	AACGCAGTCCGGAACGGGG	TGTCTAGGCAGCTGATCATGCCA	64	148
*Prl4a1*	GGAGACCATAGAGAAGATT	GCAAGAGTTCCAATTCAGA	55	89
*Ctsl*	TTTGCAGACTTCTTGTGCGC	GAGCGTGAGAACAGTCCACA	55	563
*Gapdh*	GTCGTGGAGTCTACTGGTGTC	GAGCCCTTCCACAATGCCAAA	60	240

### Probe preparation

Whole chromosome painting probes for mouse Chr. 13 were kindly provided by Dr. Michael Speicher (Institute of Human Genetics Medical University of Graz, Graz, Germany). These probes were labeled with biotin by degenerated oligonucleotide primed (DOP)—PCR. DNA FISH probes for the Prl gene cluster were labeled with dinitrophenyl (DNP), and probes for the Cts gene cluster were labeled with digoxigenin (Dig) by the Nick Translation kit using bacterial artificial chromosome DNA clones. Prl gene cluster: RP23-189A16, Cts gene cluster: RP-356J22. The labeled DNA probes were suspended in 50% formamide/10% dextransulfate/2×SSC.

### 3D-FISH

TS cells were cultured on coverslips. Cells were fixed in 4% paraformaldehyde (PFA) for 10 min and then rinsed in PBS. For permeabilization, coverslips were soaked in 0.5% saponin and 0.5% TritonX-100 in PBS for 20 min at room temperature (RT). After permeabilization, samples were incubated in 20% glycerol in PBS for at least 30 min at RT and then were soaked in liquid nitrogen until completely frozen, followed by freezing/thawing in 20% glycerol in PBS five times. Coverslips were rinsed in PBS, treated with 0.1 N HCl for 10 min, and then incubated in 0.002% pepsin in 0.01 N HCl at 37°C for 3 min followed by washing in 0.05 M MgCl_2_ in PBS at RT. Samples were rinsed in PBS and fixed in 1% PFA in PBS for 10 min. Coverslips were rinsed in PBS and in 2×saline sodium citrate (SSC), and then transferred into 50% formamide/2×SSC at 4°C until hybridization. The labeled DNA probes were denatured at 80.5°C for 6 min. After the denatured probes were loaded on microscopic slides, each coverslip was turned upside down on the probe drop and then covered with Fixogum rubber cement. Samples were denatured at 75°C for 4 min and hybridized in a water bath in a metal box at 37°C for 3 days. Samples were washed first in 2×SSC and then in 0.1×SSC at 62.5°C three times for 5 min each. Coverslips were rinsed in 4×SSC with 0.2% Tween-20 (SSCT) and blocked with 5% BSA in 4×SSCT at 37°C for 30 min. Samples were incubated with antibodies in 5% BSA in 4×SSCT. After washing in 4×SSCT, samples were counterstained with DAPI, after which VECTASHIELD Mounting Medium (Vector Laboratories, Burlingame, CA, USA) was applied for mounting. The antibodies are listed in Table 2.

**Table 2:** Antibodies used for western blotting and immunostaining.

	Source	Cat#	Dilution rate
Mouse anti-mouse-Lamin B1	Santa Cruz	sc-374015	IF (1:1000) WB(1:1000)
Rabbit anti-human-Lamin A	Abcam	ab26300	IF (1:1000)
Rabbit anti-H3K9me2	Active Motif	39239	IF (1:1000)
Rabbit anti-H3K9me3	Active Motif	39157	IF (1:1000)
Rabbit anti-H3K27me3	Active Motif	39766	IF (1:1000)
Mouse anti-mouse-cathepsinL	Santa Cruz	sc-390367	WB(1:1000)
Rabbit anti-human-cleaved caspase-3	Cell Signaling Technology	9661	IF (1:400)
Rabbit anti-human-G3PDH	R&D Systems	2275-PC-100	WB(1:1000)
Donkey-anti-mouse-Alexa488	Life Technologies	A21202	IF (1:300)
Donkey-anti-rabbit-Alexa594	Life Technologies	A21207	IF (1:300)
Sheep-anti-mouse-HRP	GE Healthcare	NA931	WB(1:10000)
Donkey-anti-rabbit-HRP	GE Healthcare	NA934	WB(1:10000)
Avidin-Cy5	Jackson Immuno Reseach Laboratories	003-170-083	IF (1:200)
Rabbit anti-DNP	Merck/Sigma-Aldrich	D-9656	IF (1:300)
Mouse-anti-Dig	Merck/Sigma-Aldrich	D-8156	IF (1:300)
Goat-anti-avidin-Bio	Vector Laboratories	BA-0300	IF (1:200)
Goat-anti-rabbit-Alexa488	Invitrogen	A-11008	IF (1:300)
Sheep-anti-mouse-Cy3	Jackson Immuno Reseach Laboratories	515-165-062	IF (1:300)

### Immunofluorescence

TS cells were cultured on microscope slides. Cells were fixed in 4% PFA for 20 min, rinsed briefly with PBS, and permeabilized for 10 min with 0.25% Triton X-100/PBS at RT. Prior to incubation with primary antibodies overnight at 4°C, the cells were blocked with 3% bovine serum albumin/PBS for 1 h at RT. The cells were washed with PBS and then incubated with secondary antibodies for 1 h in the dark at RT. After washing with PBS, Fluoro-KEEPER Antifade Reagent, Non-Hardening Type with DAPI (Nacalai Tesque) was applied for mounting. Placentas were collected from 2 or 3 C57BL/6N females at 14.5, 16.5, and 19.5 days post coitum (dpc) and fixed in 4% PFA overnight at 4°C. Samples were soaked in 10, 20, and 30% sucrose solution and were frozen in an embedding OCT compound (Sakura Finetek, Torrance, CA, USA) at -80°C. Cryosections (8 μm thick) were placed on slides. Antigen retrieval was done by microwave boiling in 10 mM citrate buffer for 8 min. Slides were blocked with 10% FBS/PBS for 1 h at RT and incubated with primary antibodies overnight at 4°C. After washing with 0.025% Triton X-100/PBS, the slides were incubated with secondary antibodies for 2 h in the dark at RT. The slides were then washed and stained with DAPI, after which VECTASHIELD Mounting Medium (Vector Laboratories) was applied for mounting.

The antibodies are listed in Table 2.

### Live/Dead cell staining

Cell viability was determined with the Live/Dead Cell Staining Kit Ⅱ (PromoKine, Heidelberg, Germany) according to the manufacturer’s protocol. Live cells were stained by Calcein AM (green fluorescence) and dead cells were stained by ethidium homodimer Ⅲ (red fluorescence). To measure the nuclei of live cells, the nuclei were stained by Hoechst 33342 at the same time. Images were obtained with fluorescence microscopy.

### Fluorescence microscopy analysis

Immunofluorescence images were obtained by fluorescence microscopy (BZX-700; Keyence, Osaka, Japan) and confocal laser scanning microscopy (LSM710; Carl Zeiss, Oberkochen, Germany), equipped with alpha Plan-apochromat 100x/1.46 oil or with Plan-apochromat 63x/1.40 oil and Plan-apochromat 40x/1.40 oil. Images were analyzed using LSM software ZEN 2011 and ImageJ software (WS Rasband, National Institutes of Health, Bethesda, MD, USA; https://imagej.nih.gov/ij).

### Signal intensity

The signal intensity of LMNB1 was semiquantitatively graded into three groups: high, signal intensity was more than 50% than that of control (Day 0 TS cells); low, signal intensity was between 20 and 50% that of control; depleted, signal intensity was less than 20% that of control (Fig 3C).

### Transmission electron microscopy

The samples were prepared and observed by Tokai Electron Microscopy, Inc. (Nagoya, Japan). The cells were fixed with 2% PFA and 2% glutaraldehyde (GA) in 0.1 M phosphate buffer (PB) pH 7.4 at RT and then were put into a refrigerator for 30 min to decrease the temperature to 4°C. Thereafter, they were fixed with 2% GA in 0.1 M PB overnight at 4°C. After fixation, the samples were washed three times with 0.1 M PB for 30 min each and were postfixed with 2% osmium tetroxide in 0.1 M PB for 1 h at 4°C. The samples were dehydrated in graded ethanol solutions (50, 70, 90, and 100%). The schedule was as follows: 50 and 70% for 5 min each at 4°C, 90% for 5 min at RT, and 3 changes of 100% for 5 min each at RT. The samples were transferred to resin (Quetol-812; Nisshin EM Co., Tokyo, Japan) and were polymerized for 48 h at 60°C. The polymerized resins were ultrathin-sectioned at 70 nm with a diamond knife using an ultramicrotome (Ultracut UCT; Leica, Vienna, Austria), and the sections were mounted on copper grids. They were stained with 2% acetate at RT for 15 min, washed with distilled water, and secondary-stained with Lead stain solution (Sigma-Aldrich Co., Tokyo, Japan) at RT for 3 min. The grids were observed by a transmission electron microscope (JEM-1400Plus; JEOL Ltd., Tokyo, Japan) at an acceleration voltage of 100 kV. Digital images (3296×2472 pixels) were taken with a CCD camera (EM-14830RUBY2; JEOL Ltd.).

### Western blotting

TS cells were lysed on ice in SDS lysis buffer containing 62.5 mM Tris-HCl (pH 6.8), 2% SDS, 10% glycerol, 2.5% β-mercaptoethanol, and bromophenol blue. Cell lysates were boiled for 5 min at 95°C and were fractionated by SDS-PAGE using 10% polyacrylamide gel. Proteins were transferred to PVDF membranes (Roche Diagnostics, Mannheim, Germany), blocked with 5% nonfat milk in wash buffer (25 mM Tris-HCl, 150 mM NaCl, and 0.05% Tween-20) for 1 h, and incubated with primary antibodies overnight at 4°C. Membranes were washed and incubated with horseradish peroxidase-linked secondary antibodies for 1 h at RT. Signals were detected with an ECL Prime Western Blotting Detection Reagent and visualized by a luminescent image analyzer (LAS-3000, Fujifilm, Tokyo, Japan). Samples consisting of 1×10^4^ cells were used for the detection of proteins. The expression levels of LMNB1 and GAPDH were quantified using ImageJ (NIH).

### Time-lapse imaging

Time-lapse imaging was performed using microscopy with an incubation system (BZX-700; Keyence). TS cells cultured without FGF4 for 6 days were used to obtain images at 15-min intervals for 3 days.

### Statistical analysis

Results are presented as means ± SE of three independent experiments unless otherwise stated. Statistical analysis was performed with Student’s *t*-test or the χ2 test. In Fig 6B, percentages of dead TGCs were analyzed by one-way analysis of variance (ANOVA) followed by Dunnett’s test using JMP software (SAS Institute. Cary, NC, USA). Statistical significance was defined as follows: *, P < 0.05. **, P < 0.01. ***, P<0.001. In the case ofχ2 test, different letters indicate significant difference (P < 0.05).

## Results

### Chromosome organization in TGCs

**Fig 1:**
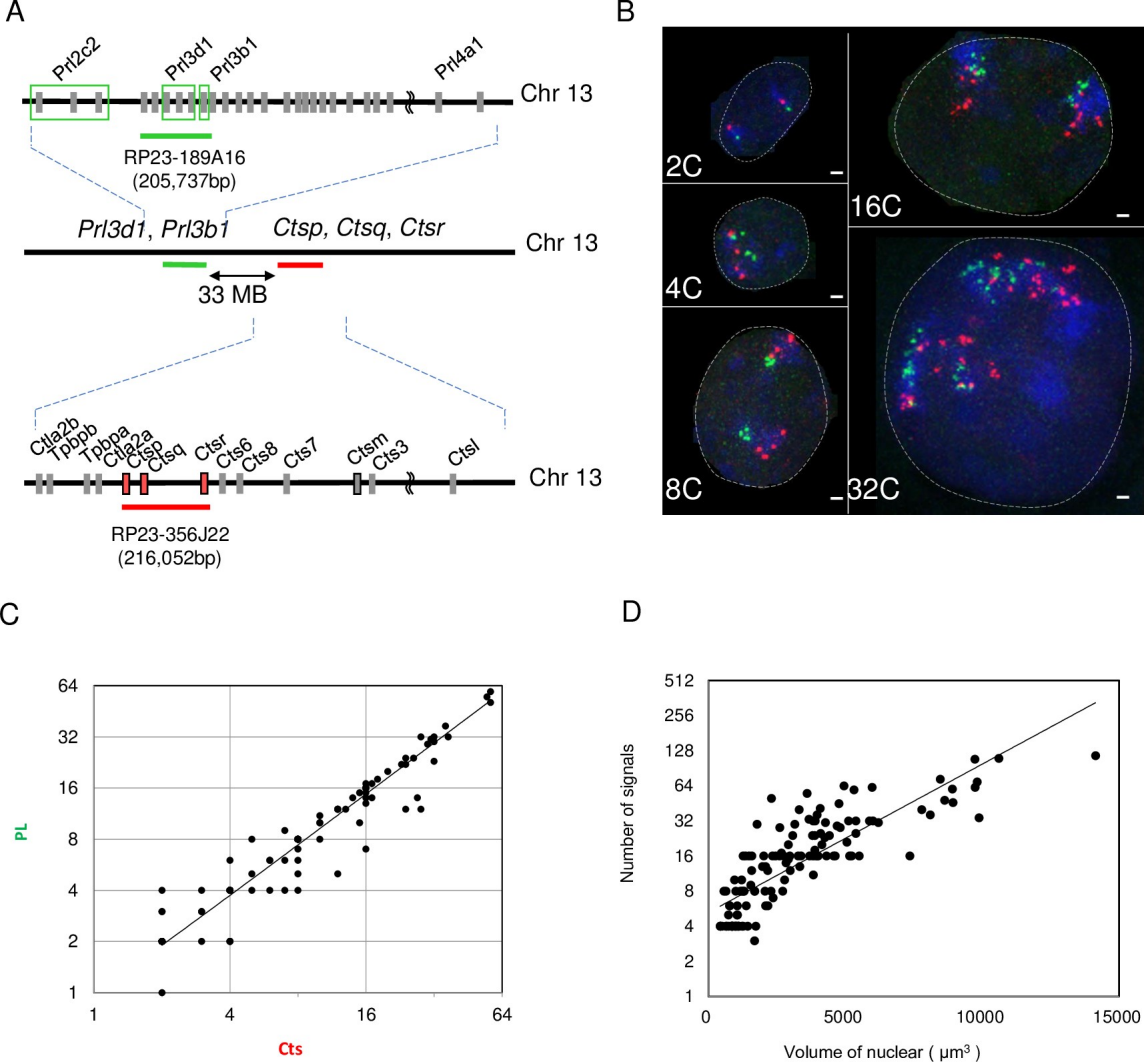
Visualization of Prl and Cts loci in TGCs. (A) Scale diagram of the Prl and Cts gene regions on chromosome 13. BAC clones to detect Prl and Cts loci are shown by green and red lines, respectively. (B) Arrangement of Prl and Cts loci in 2C TSC and 4C, 8C, 16C, and 32C TGCs. Bars mean 5 μm. (C) Relationship between the numbers of Prl and Cts signals in a TGC nucleus. (D) Relationship between nuclear volume and numbers of Prl and Cts signals.

To clarify the relationship between DNA replication and nuclear volume in TGCs, PL and Cts loci were detected by a DNA probe prepared from specific bacterial artificial chromosome (BAC) clones (BAC clones RP23-189A16 and RP23-356J22, respectively; purchased from Advanced Geno Techs, Ibaraki, Japan) (Fig 1A). In 2C nuclei, two loci of PL and Cts were detected. During TGCs formation, the numbers of these two loci increased in parallel with each other (Fig 1B and 1C) and depended on nuclear size (Fig 1D). In some cases, these two loci were spread across the nuclei (S1A Fig). To further analyze the chromosome organization in TGCs, the chromosome territories were observed by whole-chromosome painting probes for chromosomes 5, 12, and 13. In TGCs, compact chromosomes 5, 12, and 13 were observed in nuclei (S1B Fig). In some cases, 1) compact chromosomes 12 and 13 and dispersed chromosome 5 (S1C Fig) were observed, as were 2) compact chromosomes 5 and 12 and dispersed chromosome 13 (S1D Fig) and 3) dispersed chromosomes 5, 12, and 13 (S1E Fig). These results indicated that some chromosomes are dispersed in the nuclei of TGCs.

### Localization of nuclear peripheral heterochromatin

**Fig 2:**
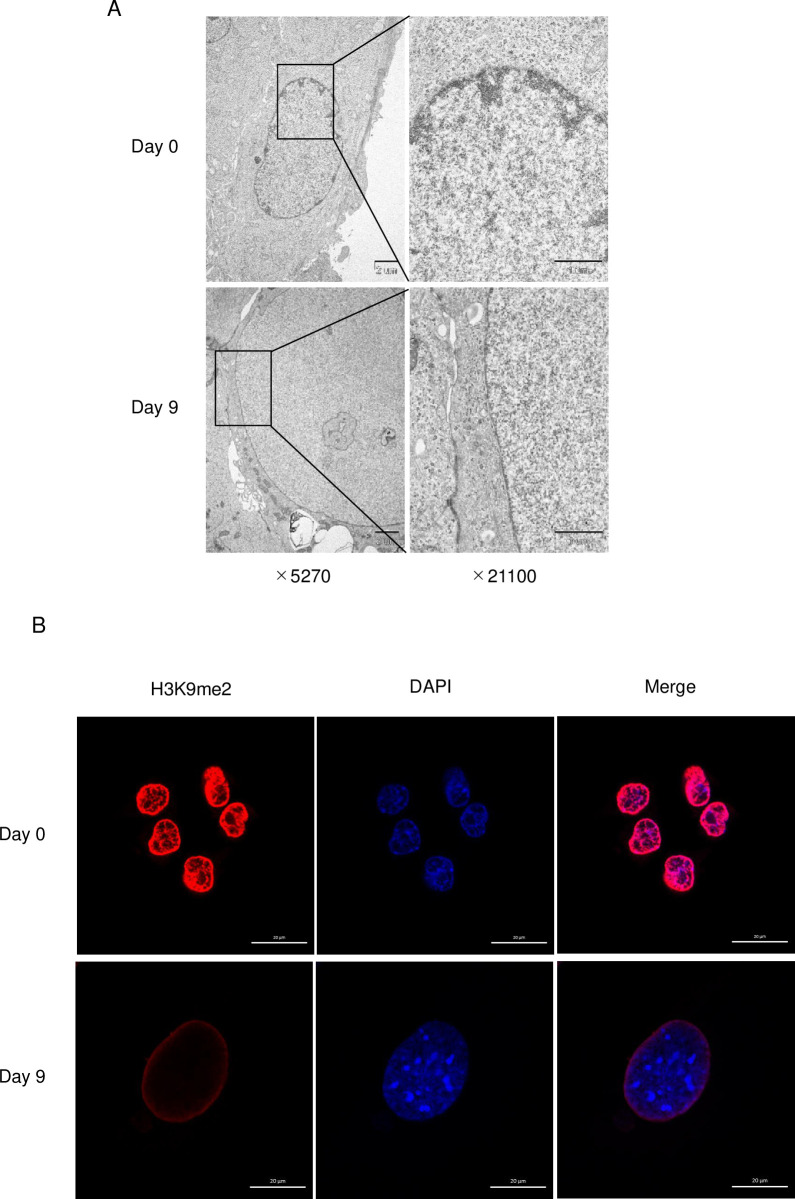
Nuclear structures of TSCs and TGCs. (A) Electron micrographs of TSC (Day 0) and TGC (Day 9) nuclei. (B) Localization of H3K9me2 in TSC (Day 0) (n = 341) and TGC (Day 9) (n = 226). Bars mean 20 μm.

To analyze the nuclear organization in TGCs, electron microscopic observation of TSCs and TGCs was carried out at day 9 after differentiation. In TSCs, heterochromatin was distributed at the periphery of the cell nucleus, but in TGCs, heterochromatin was absent at the periphery (Fig 2A). According to a recent report, H3K9me2 was an evolutionarily conserved marker of peripheral heterochromatin [28]. In TSCs, H3K9me2 was localized at the periphery of the cell nucleus. However, H3K9me2 was localized at nuclear periphery of 53% of TGCs, and 12.8% of them showed the loss of H3K9me2 at the periphery (Fig 2B). On the other hand, some TGCs showed the loss of H3K9me2 at the periphery (Fig 2B). These results indicated that the localization of H3K9me2 marked heterochromatin was disturbed at the TGCs nuclear periphery.

### Loss of Lamin B1 during TGC formation

**Fig 3:**
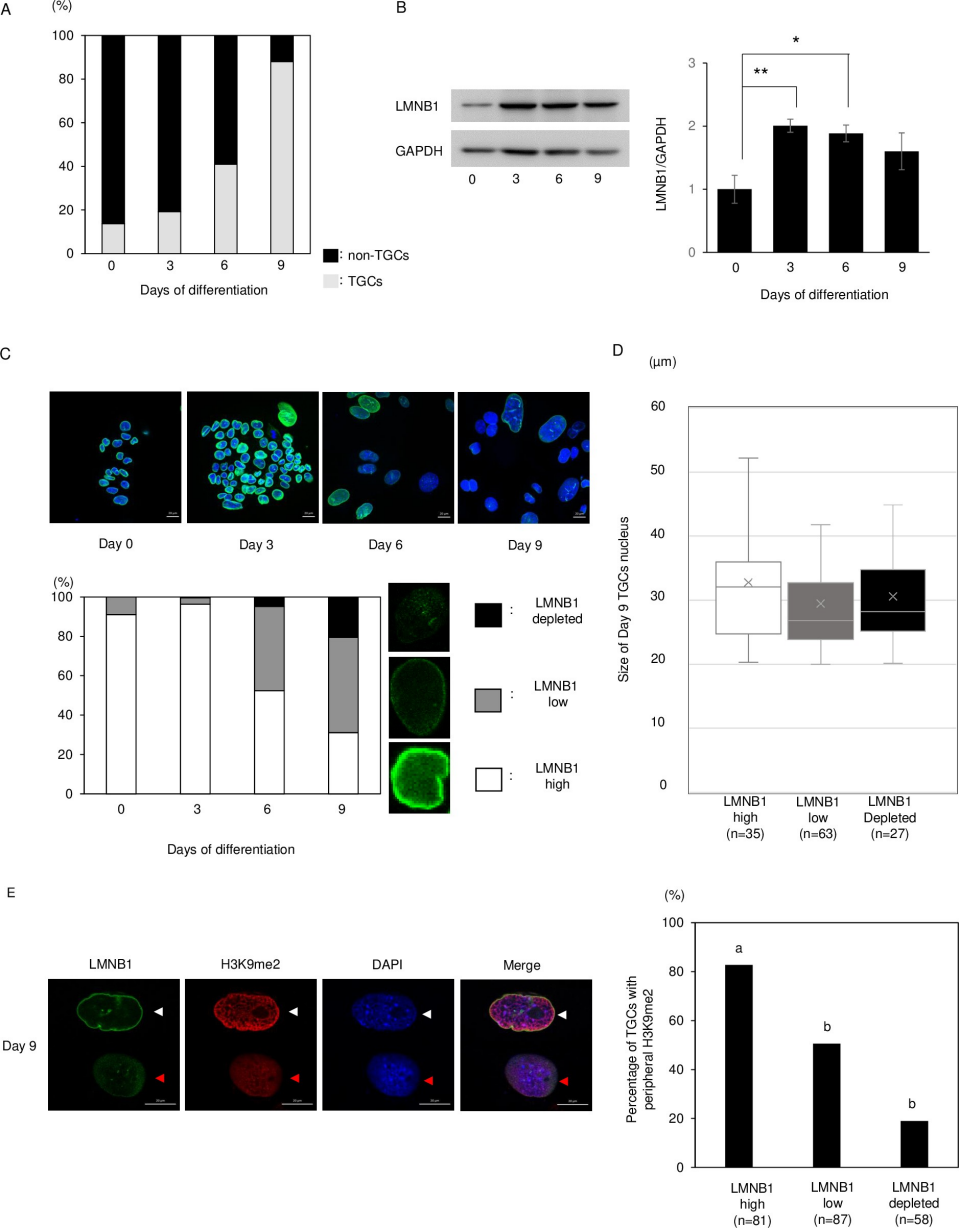
Localization of LMNB1 during TGC formation. (A) Percentages of TGCs at days 0–9 after differentiation. The percentage of TGCs dramatically increased from days 6 to 9. (B) Western blot analysis of LMNB1 at days 0–9 after differentiation. The amount of LMNB1 protein peaked at day 3 and then gradually decreased. The data are represented as means ± SE (n = 5). Significance was determined by the t-test as a comparison between day0 and each day after differentiation. *Indicates P < 0.05, **indicates P < 0.01. (C) Localization of LMNB1 at days 0–9 after differentiation. Although almost all cells were LMNB1-high at day 0, 50% and 20% were LMNB1-low and -depleted, respectively, at day 9 (n >150, each stage). (D) The size of day 9 TGCs nucleus: LMNB1 high (n = 35), low (n = 63) and depleted (n = 27). Box plots show the central 75% (boxes), median (lines in boxes), average (x marks), and range (whiskers). (E) Localization of LMNB1 and H3K9me2 at day 9 after differentiation. White arrowheads indicate LMNB1-high TGCs and red arrowheads indicate LMNB1-low TGCs. The percentage of TGCs with peripheral H3K9me2 decreased significantly in LMNB1-low and -depleted cells. Statistical analysis was performed by the χ2 test: LMNB1 high (n = 81), low (n = 87), and depleted (n = 58). Different letters indicate significant difference (P<0.05).

Chromosome territories and localization of heterochromatin marker were disturbed in *Lmnb1* silencedbDLD-1 cells had been reported [8]. Therefore, we focused on LMNB1 as a scaffolding structure of heterochromatin. In this study, we defined the cells that the nuclear size was more than 20 μm in diameter as TGCs. After the differentiation, TGCs gradually increased, and at day 9 after differentiation, about 90% of cells were TGCs (Fig 3A). LMNB1 also increased after differentiation, but the amount of protein peaked at day 3 after differentiation and decreased slightly from days 3 to 9 after differentiation despite the increase in the population of TGCs (Fig 3B). The localization of LMNB1 was detected by immunostaining. In almost all TGCs, the signal of LMNB1 was detected at nuclear periphery. TSCs had uniformly localized LMNB1 (LMNB1-high). However, the numbers of low and depleted LMNB1 cells increased after differentiation and, at day 9 after differentiation, 50% and 20% of LMNB1 cells were low and depleted, respectively (Fig 3C). The loss of LMNB1 did not depend on nuclear size (Fig 3D). These results indicated that the loss of LMNB1 in TGCs might be related to the time after induction of differentiation. These LMNB1-low and -depleted cells significantly decreased the loss of H3K9me2 at the periphery of the cell nucleus (Fig 3E). TGCs with the loss of Lamin B1 also diffused the peripheral localization of the other heterochromatin markers, H3K9me3 and H3K27me3 (S2 Fig).

### Effect of Lamin B1 on TSC gene expression

**Fig 4:**
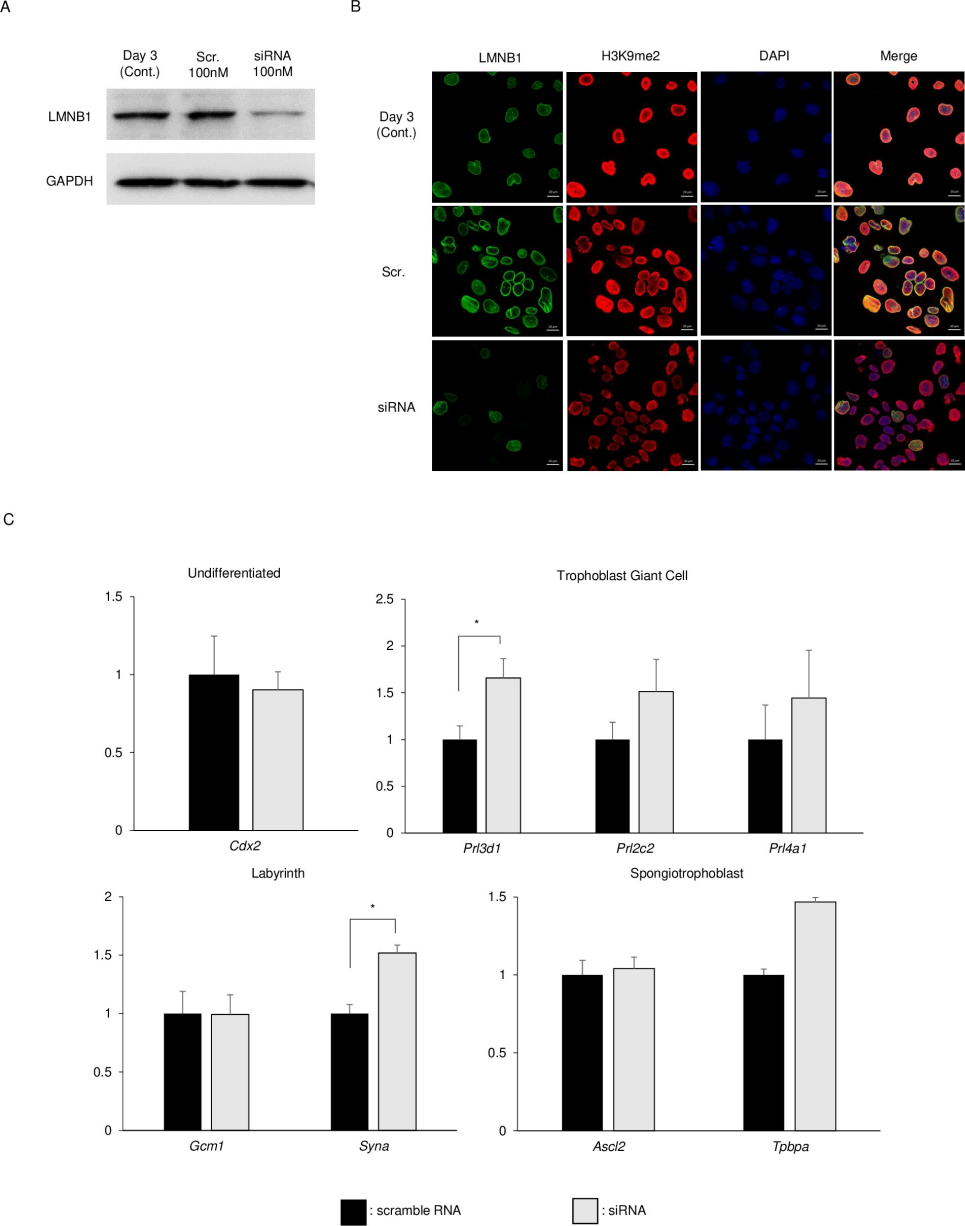
Knockdown of *Lmnb1* in TGCs by siRNA. siRNA-transfected TSCs were cultured for 3 days in TS medium without Fgf4. LMNB1 protein was then detected by western blotting (A), and the localization of LMNB1 and H3K9me2 was detected by immunostaining. (C) Expression levels of undifferentiated gene (*Cdx2*), trophoblast giant cell markers (*Prl3d1*, *Prl2c2*, and *Prl4a1*), labyrinth markers (*Gcm1* and *Syna*), and spongiotrophoblast markers (*Ascl2* and *Tpbpa*) were examined by qRT-PCR. Expression levels were normalized with *Gapdh*. The data are presented as means ± SE (n = 3). Significance was determined by the *t*-test. *Indicates P < 0.05 compared to scramble RNA.

To examine the effect of Lamin B1 on the differentiation of TSCs, *Lmnb1* knockdown (KD) TGCs were used (Fig 4A). *Lmnb1* KD TGCs lost H3K9me2 at the periphery of the cell nucleus (Fig 4B). These results indicated that chromatin organization in TGCs was dependent on LMNB1. Because the chromatin organization was changed in *Lmnb1* KD TGCs, we focused on changes in the gene expression patterns. Therefore, the expression of a TS marker gene (*Cdx2*), labyrinth marker genes (*Gcm1* and *Syna*), spongiotrophoblast marker genes (*Ascl2* and *Tpbpa*), and TGC marker genes (*Prl3d1*, *Prl2c2*, and *Prl4a1*) were analyzed. Contrary to our expectations, the expression levels of these marker genes were similar between *Lmnb1* KD and control TSCs, with two exceptions (*Syna* and *Prl3d1*), indicating that *Lmnb1* did not affect the expression of trophoblast-specific genes or the differentiation of TSCs (Fig 4C).

### Localization of Lamin B1 in placenta

**Fig 5:**
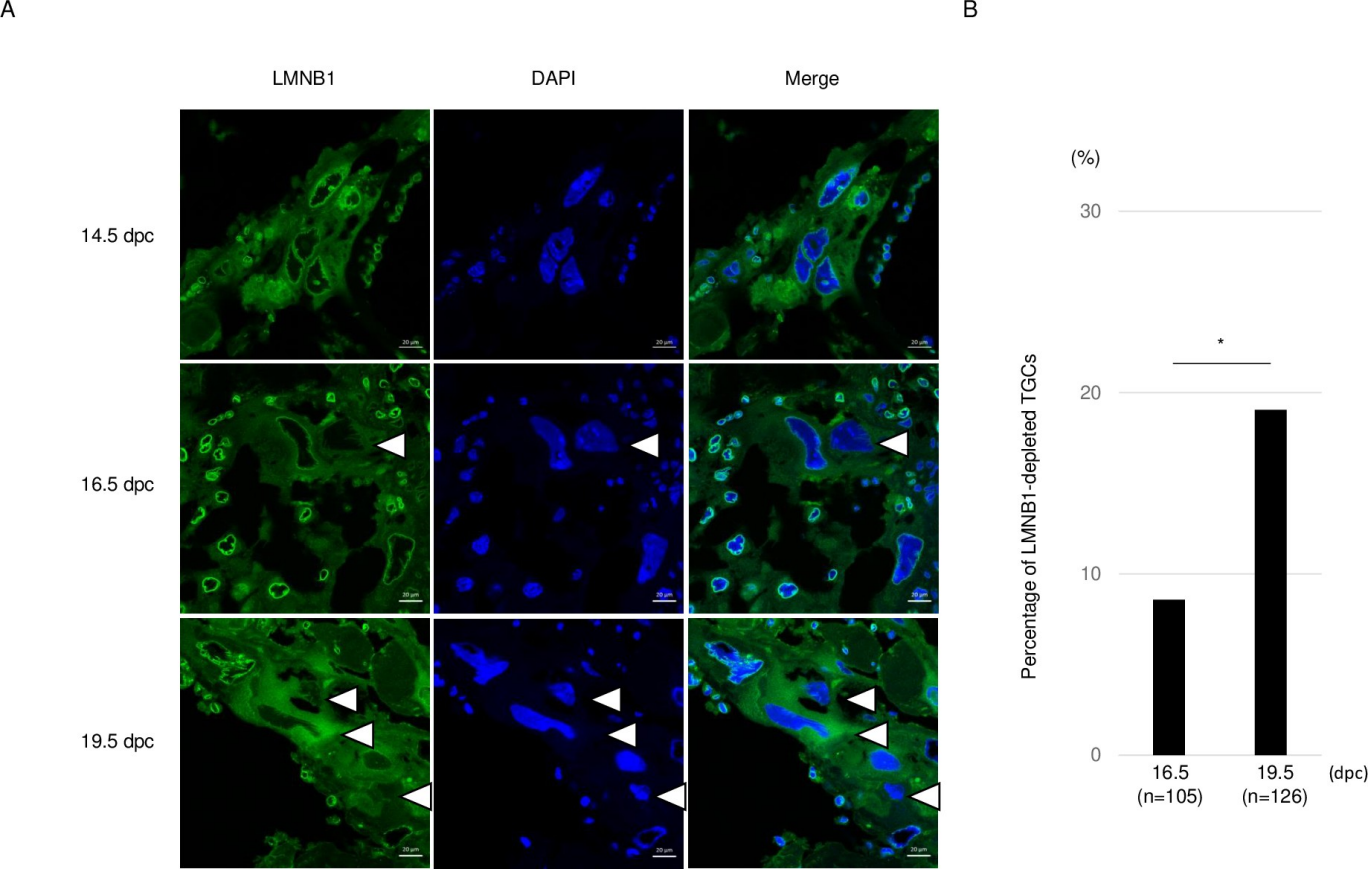
LMNB1 expression in placenta at 14.5, 16.5, and 19.5 dpc. (A) White arrowheads indicate LMNB1-depleted TGCs. LMNB1-depleted TGCs were detected at 16.5 and 19.5 dpc. (B) Percentage of LMNB1-depleted TGCs. Statistical analysis was performed by theχ2 test:16.5 dpc (n = 105) and 19.5 dpc (n = 128). *, P < 0.05.

The localization of LMNB1 in TGCs was detected in placenta at 14.5, 16.5, and 19.5 dpc (Fig 5A). At 14.5 dpc, all TGCs were LMNB1-positive. At 16.5 dpc, almost all TGCs were LMNB1-positive, but a small number of TGCs were depleted of LMNB1 (8.6%). At 19.5 dpc, the number of LMNB1-depleted TGCs was increased (19.5%) (Fig 5B). These results suggest that TGCs in placenta lost LMNB1 toward parturition.

### Morphology of TGCs cultured in vitro

The morphological changes during TSC differentiation into TGCs were observed by time-lapse imaging. After differentiation, the cells were larger during differentiation. After day 6 of differentiation, some TGCs showed explosive death (S3 Fig).

### The mechanism underlying Lamin B1 deficiency in TGCs

**Fig 6:**
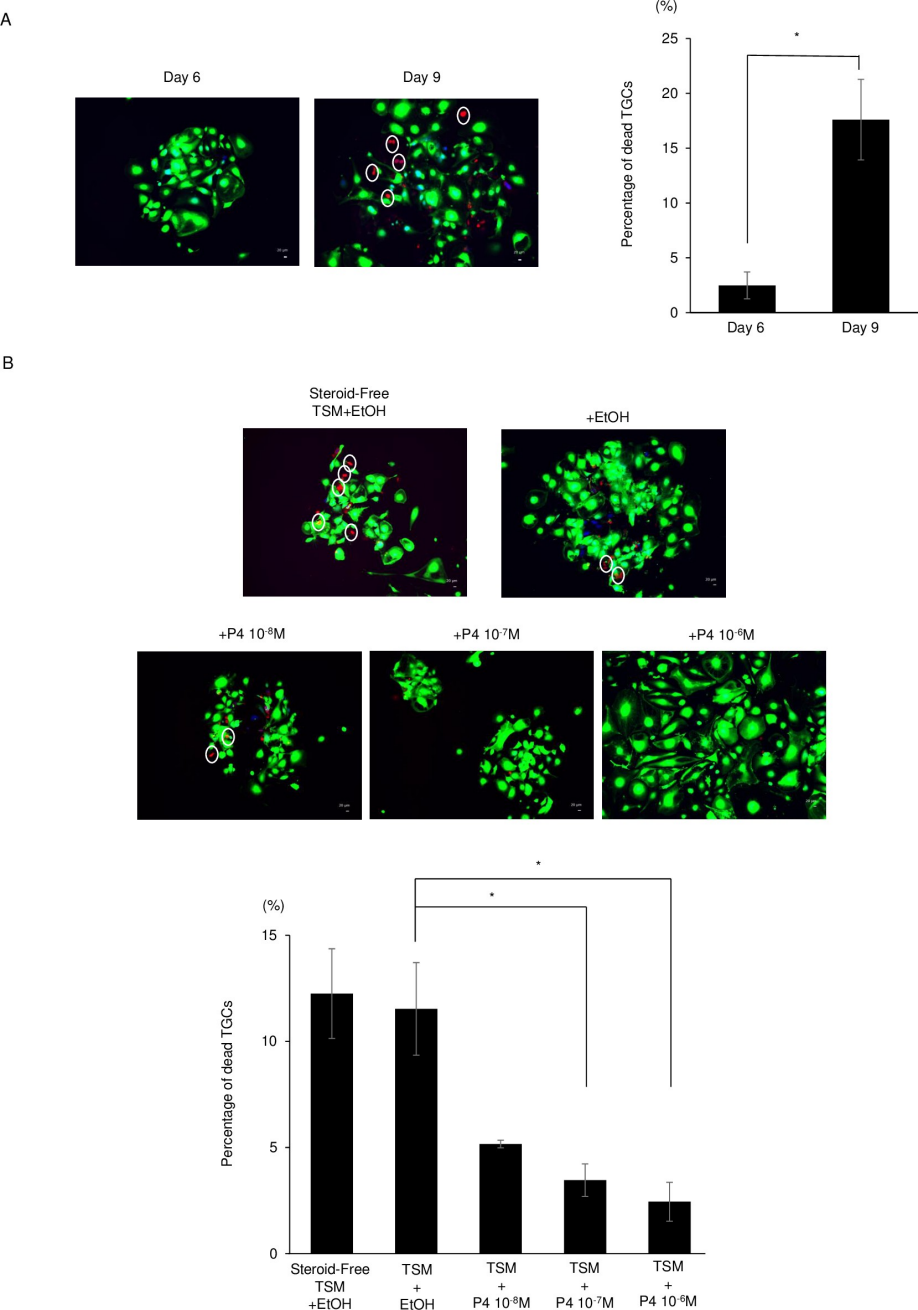
Cell viability of TGCs. (A) Cell viability was detected with the Live/Dead Cell Staining Kit Ⅱ. Live cells were stained by Calcein AM (green) and dead cells were stained by ethidium homodimer Ⅲ (red). The percentage of dead TGCs was significantly higher at day 9 than at day 6. (B) Effect of P_4_ on the viability of TGCs. The percentages of dead TGCs decreased significantly in a P_4_-dose-dependent manner. White circles indicate dead TGC nucleus. The data are presented as means ± SE (n = 3). The percentage of dead TGCs were analyzed by ANOVA followed by Dunnett’s test. Dunnett’s test was performed as a comparison between TSM + EtOH and each concentration of P4. Statistical significance was defined as follows: *, P < 0.05.

Time-lapse imaging showed TGCs bursting after day 6 of differentiation. This indicated that TGC cell death was induced between days 6 and 9 after differentiation. Therefore, the cell viability of TGCs was analyzed. The percentages of dead cells at days 6 and 9 after differentiation were increased to 2.5% and 17.6%, respectively (Fig 6A). At the same time points, the percentage of TGCs with Lamin B1 loss increased (Fig 3C).

**Fig 7:**
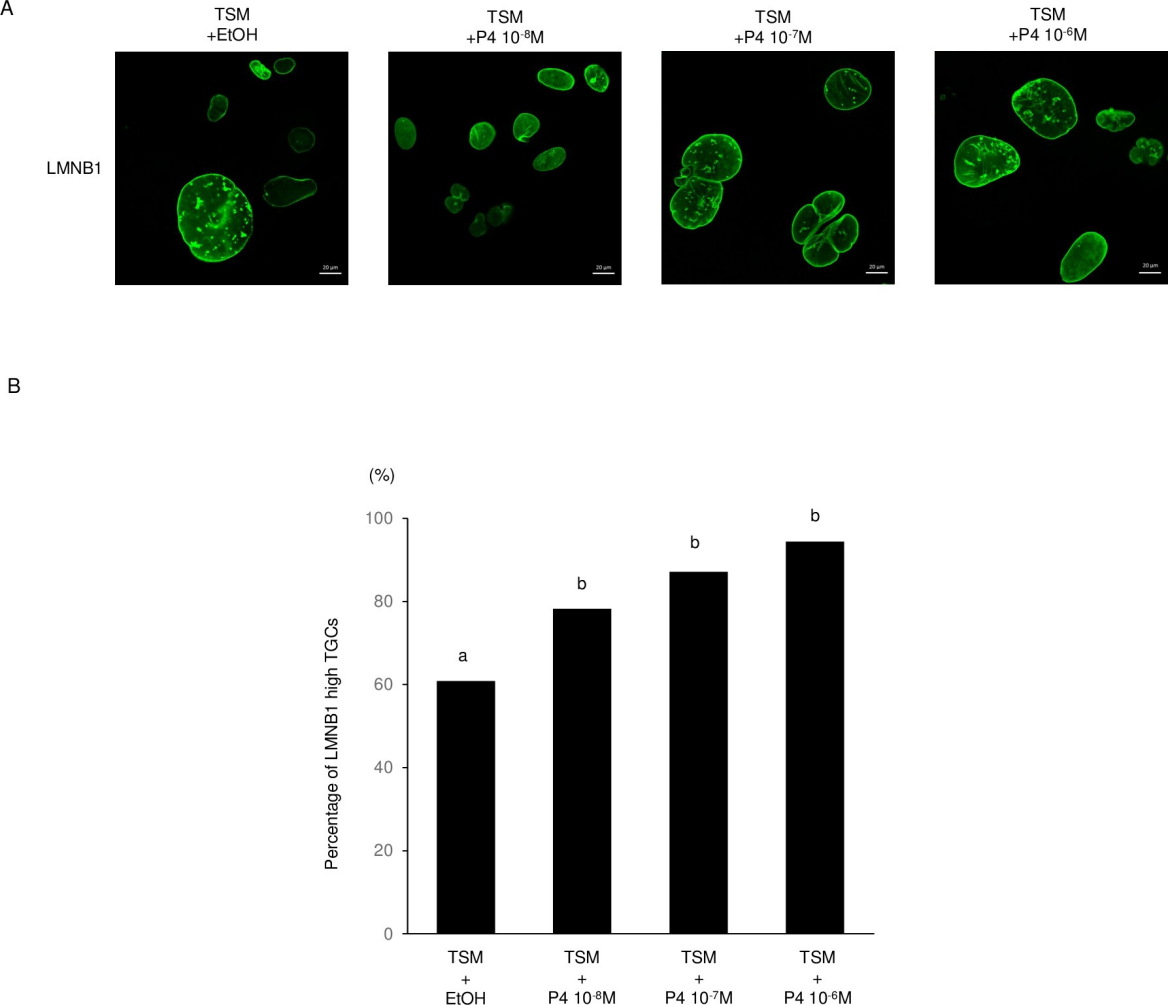
Effect of P_4_ on localization of LMNB1 in TGCs. (A) Localization of LMNB1. Bars mean 20 μm. (B) Percentages of LMNB1-high TGCs increased significantly in a P4-dose-dependent manner. Statistical analysis was performed with the χ2 test: +EtOH (n = 161), 10^-8^M (n = 231), 10^-7^M (n = 171) and 10^-6^M (n = 133). Different letters indicate significant difference (P<0.05).

In this study, Lamin B1 was disturbed in E19.5 placenta (Fig 5). In mammals, progesterone (P_4_) and estrogen (E_2_) are essential for the maintenance of pregnancy. In mice, maternal P_4_ levels were steady from E13.5 to 16.5, began to drop at E17.5, and remained low until parturition [29]. Therefore, the effect of P_4_ on the localization of Lamin B1 in TGCs was examined in vitro. When TGCs were cultured with FBS or charcoal/dextran−treated FBS (steroid-free FBS), about 12% of cells died. On the other hand, the percentage of dead TGCs cultured with 10^−6^ M P_4_ was less than 5% (Fig 6B). Moreover, the rate of LMNB1-high TGCs was significantly increased, by 10^−6^ to 10^−8^ M P_4_, in a dose-dependent manner (Fig 7). These results indicate that P_4_ may protect against Lamin B1 loss and prolongs the life and function of TGCs.

## Discussion

Mouse TSCs were established from the polar TE of blastocysts. TSCs were differentiated into labyrinth, spongiotrophoblast, and TGCs and terminally into TGCs [30]. Gene expression analyses have revealed that differentiated TSCs show time-specific expression of differentiated markers [27,31,32]; e.g., *Ascl2* (a spongiotrophoblast-specific gene) and *Gcm1* (a labyrinth-specific gene) express transiently within a few days after differentiation. After that, expression levels of *Tpbpa* (a spongiotrophoblast-specific gene) and *Prl3d1*, *Prl3b1*, *Prl2c2*, and *Prl4a1* (TGC-specific genes) increase gradually. *Prl3d1* and *Prl3b1* express up to day 14 after differentiation [32]. By day 6 after differentiation, higher ploidy cells (>8N) started to appear [30]. Therefore, TGCs express specific genes for at most 7 days. Despite the many reports on gene expression, the mechanisms underlying TGC-specific gene expression have been unclear. To clarify those mechanisms, we focused on the nuclear structure.

Mouse chromosome 13 contains three gene clusters: prolactin (Prl), serpins (Ser), and cathepsins (Cts). These regions are known to be important for placentation and pregnancy [7]. Prl genes have two separate clusters. The mini Prl cluster (3 Prl genes) is located approximately 14 Mb upstream of the big Prl cluster (23 Prl genes) [33]. The Ser cluster (15 Ser genes) and the Cts cluster (12 Cts genes) are located approximately 7 Mb and 34 Mb downstream of the big Prl cluster, respectively. Osborne et al. [34] showed that mouse anemic spleen erythroid cells expressed *Hbb* and *Eraf*. Although these two gene loci were 25 Mb apart from each other, they were close together and occupy the same RNAP Ⅱfocus. During TGC formation, the Prl and Cts gene clusters were activated. Therefore, we analyzed whether the Prl and Cts loci were close to the TGC nuclei. Contrary to our expectations, these loci were not close together but were spread apart, indicating that TGC-specific gene expression was not related to the accumulation of transcription-activated loci. Interestingly, in some TGCs, the loci were widely dispersed in the nuclei. In the interphase nuclei of vertebrates, individual chromosomes occupy distinct territories [35,36]. However, spreading chromosomes were observed in TGC nuclei. Moreover, the transmission electron microscope images showed that TGCs had decreased levels of nuclear peripheral heterochromatin. According to a recent report, H3K9me2 is an evolutionarily conserved marker of peripheral heterochromatin and is required for nuclear peripheral localization of chromatin [28]. However, some TGCs showed the loss of H3K9me2 at the periphery of the cell nucleus.

The nuclear lamina is a major structural element of the nuclear envelope in interphase nuclei. In mammals, nuclear lamina is composed of A-type lamins (Lamin A and C) and B-type lamins (Lamin B1 and B2). Lamins A and C are the products of alternative splicing of *Lmna*, whereas Lamin B1 and B2 are encoded by *Lmnb1* and *Lmnb2*, respectively [11]. Among these genes, LMNB1 plays an important role in maintaining chromatin compaction in the nucleus [8]. Also, a correlation between H3K9me2 and Lamin B1 in mouse embryonic stem cells was reported [28]. Thus, we focused on LMNB1 as a scaffolding structure of heterochromatin in TGC nuclei. As a result, the loss of LMNB1 in TGC nuclei was observed from day 6 after differentiation. In addition, LMNB1-loss TGCs and *Lmnb1* KD TGCs diffused the peripheral localization of the heterochromatin marker H3K9me2. These results indicate that the loss of LMNB1 causes loose heterochromatin at the periphery of the TGC nucleus. Similarly, it has been reported that TGCs form a loose chromatin structure during differentiation [37].

Because the chromatin organization was changed in *Lmnb1* KD TGCs, we focused on changes in the gene expression patterns. As a result of gene expression analysis, *Prl3d1*, a TGC-specific expressed gene, was expressed at significantly higher levels and *Prl2c2* and *Prl4a1* were expressed at slightly higher levels in *Lmnb1*-KD TGCs. *Syna* also exhibited significantly higher expression in *Lmnb1*-KD TGCs. However, *Cdx2* (an undifferented TS marker gene), *Gcm1* (a labyrinth-specific gene), and *Ascl2* (a spongiotrophoblast-specific gene) showed no significant differences in expression levels. Lamin B1−loss mouse lung epithelial (MLE12) cells were upregulated to fourfold the expression level of *Ret* and *Gfra1* [38], indicating that Lamin B1 loss induced gene expression. In the present study, upregulation of TGC-specific genes was not so drastic compared with that of TGCs differentiated in vitro (S4 Fig, [27]). Therefore, Lamin B1 loss in TGCs did not induce specific gene expression remarkably.

In general, nuclear lamina loss occurs at the M-phase during mitosis, cell senescence, and cell death. The M-phase does not occur during endoreduplication and thus differentiation into TGCs occurs. Lamin B1 loss is a biomarker of cell senescence and occurs upon activation of either the p53 or pRB pathway [14]. However, TGC differentiation requires the downregulation of p53 and RB [39]. Furthermore, senescent cells form spotty patterns of heterochromatic domains called senescence-associated heterochromatin foci (SAHF) [40], but TGCs do not. Thus, we focused on trophoblastic cell death. In this study, time-lapse and live/dead staining experiments show that TGC cell death is induced at the time of LMNB1 depletion. These results indicated that the depletion of LMNB1 is involved in TGC cell death. In murine placenta, TGC cell death increases after 15.5 dpc [23]. In the present study, TGCs with LMNB1 loss were detected at 16.5 and 19.5 dpcs in placenta, indicating that LMNB1 loss might be correlated with TGC cell death. Usually, the disruption of the nuclear lamina structure is observed in the process of apoptosis. However, TGCs escape from apoptosis by p21/Cip1 [4]. Cleaved caspase-3, a major apoptosis factor, is at a low level in late gestation (15.5–18.5 dpcs) [23]. In the present study, cleaved caspase-3 was not detected in TGCs with LMNB1 loss at day 9 after differentiation (S5 Fig). These results indicate that the loss of LMNB1 in TGCs may occurr in the process of caspase-independent programmed cell death. Recently, nurse cells in *Drosophila melanogaster* were eliminated through a caspase-independent and nonapoptotic developmental death during the late stages of oogenesis [19]. During this process, the lysosomal cathepsin L (Flybase: CP1, cysteine protease-1) [41] facilitates Lamin degradation [19]. In the present study, *Cathepsin L* mRNA was expressed in TSCs at days 0, 2, and 4 after differentiation, but active cathepsin L was detected at days 6 and 9 after differentiation (S6 Fig). Therefore, LMNB1 degradation in TGCs might be promoted by cathepsin L activation. In in vivo TGCs, cell death increased after 15.5 dpc [23]. Also after 15.5 dpc, the circulating maternal levels of progesterone were decreased [29]. These results indicate that progesterone may be key to the lifespan of TGCs. Estrogen and progesterone are necessary for implantation and maintenance of pregnancy in mammals. In mice, progesterone increased at day 3 of pregnancy prior to implantation. Until 16.5 dpc, progesterone was at a high level, with concentrations of 15–20 ng/ml. After 16.5 dpc, progesterone suddenly decreased to concentrations of less than 5 ng/ml at 18.5 dpc [29]. In the present study, progesterone was added to TGCs at final concentrations of 10^−7^ and 10^−8^ M, which resembled the concentrations at 16.5 and 18.5 dpcs, respectively. Also, the concentrations of progesterone in FBS and steroid-free FBS used in this study were about 30 pg/ml (10^-12^M) and 2.0 pg/ml (6.7×10^-14^M), respectively (analyzed by ELISA). Therefore, TGCs were cultured in TS medium with progesterone, which came from FBS less than 2×10^-13^M. As a result, the rate of cell death decreased in a progesterone-dose-dependent manner. Although TGCs gradually increased after differentiation, the percentage of dead TGCs increased from day 6 to day 9 after differentiation. These results indicate that the low level of progesterone in vitro did not affect the formation of TGCs but did affect their maintenance.

Also, the percentage of LMNB1-high TGCs increased in a progesterone-dose-dependent manner. TGCs with Lamin B1 loss were observed in placenta during late gestation, while progesterone decreased at the same time [29] and TGC cells died [23]. In a recent report, an absence of Lamin B1 causes nuclear membrane ruptures and cell death [21]. Although the relationship between progesterone and the Lamin B1 degradation factor is unclear, it is thought that the loss of Lamin B1 observed in the process of TGC cell death was caused by a deficiency of progesterone.

In conclusion, the present study demonstrated that the number of Lamin B1 loss TGCs was increased during the differentiation of TSCs. These Lamin B1 loss TGCs diffused peripheral localization of the heterochromatin marker H3K9me2 in nuclei. Furthermore, the death of TGCs also increased during the differentiation. The Lamin B1 loss and the cell death in TGCs were reduced by progesterone indicating that progesterone promotes the longevity of TGCs.

## Supporting information

S1 Fig Visualization of Prl and Cts loci and mouse chromosome 5, 12, and 13 in TGCs. (A) Prl and Cts loci were spread in TGC nuclei. (B-E) FISH chromosome painting of chromosomes 5 (blue), 12 (green), and 13 (red) in TGCs. Bars mean 5 μm.
(TIF)

S2 Fig Immunostaining of LMNB1 and epigenetic marker of heterochromatin at day 9 after differentiation. Fluorescence of H3K9me3 (top) and that of H3K27me3 (bottom) were weakened in LMNB1-loss TGCs (arrowheads). Bars mean 20 μm.
(TIF)

S3 Fig Time-lapse imaging of TGCs cultured in vitro. Imaging was carried out from days 6 to 9 after differentiation.
(AVI)

S4 Fig qRT-PCR analysis of a TGC-specific gene (*Prl3d1*) in TS cells after differentiation. Expression levels were normalized with Gapdh. The data are presented as means ± SE (n = 3).
(TIF)

S5 Fig Immunostaining of LMNB1 and cleaved caspase-3 as apoptotic marker. Cleaved caspase-3 was not detected in LMNB1-loss TGCs (arrowheads). To induce apoptosis, RO-3306 was added to NIH3T3 cells for 72 h at a final concentration of 10–5 M. Bars mean 20 μm.
(TIF)

S6 Fig Expression analysis of Cts family genes. (A) RT-PCR analysis of Cts family genes. (B) Western blot analysis of CTSL protein in TS cells after differentiation. Mature CTSL increased dramatically from day 6 after differentiation.
(TIF)

S1 Raw images (PDF)
