# Supplementary figures and images for: Progesterone depletion results in Lamin B1 loss and induction of cell death in mouse trophoblast giant cells
Source: PLoS One. 2021 Jul 14;16(7):e0254674. doi: 10.1371/journal.pone.0254674 (PMC8279370; doi:10.1371/journal.pone.0254674)

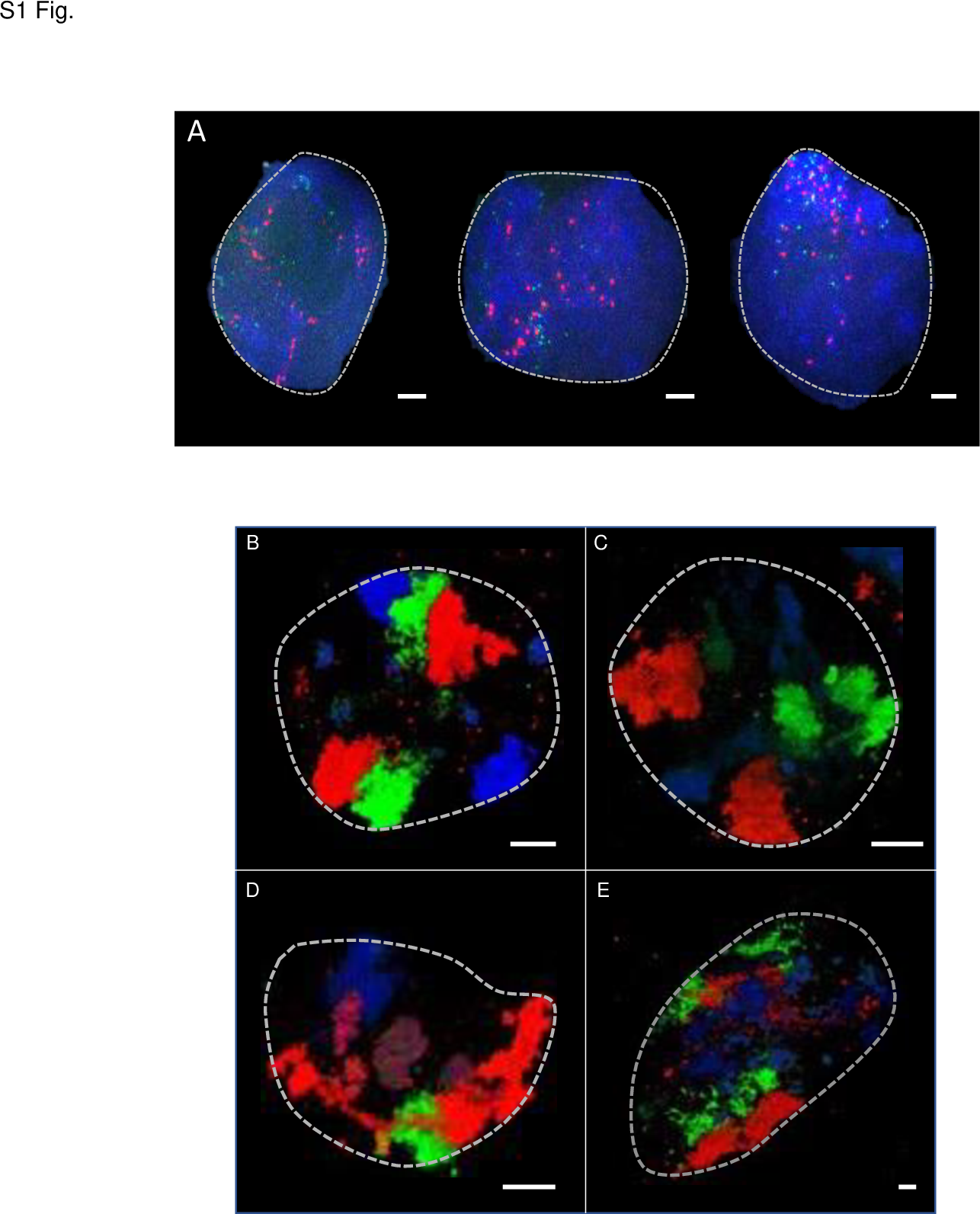

Supplement: S1 Fig — (A) Prl and Cts loci were spread in TGC nuclei. (B-E) FISH chromosome painting of chromosomes 5 (blue), 12 (green), and 13 (red) in TGCs. Bars mean 5 μm. (TIF) [file pone.0254674.s001.tif]

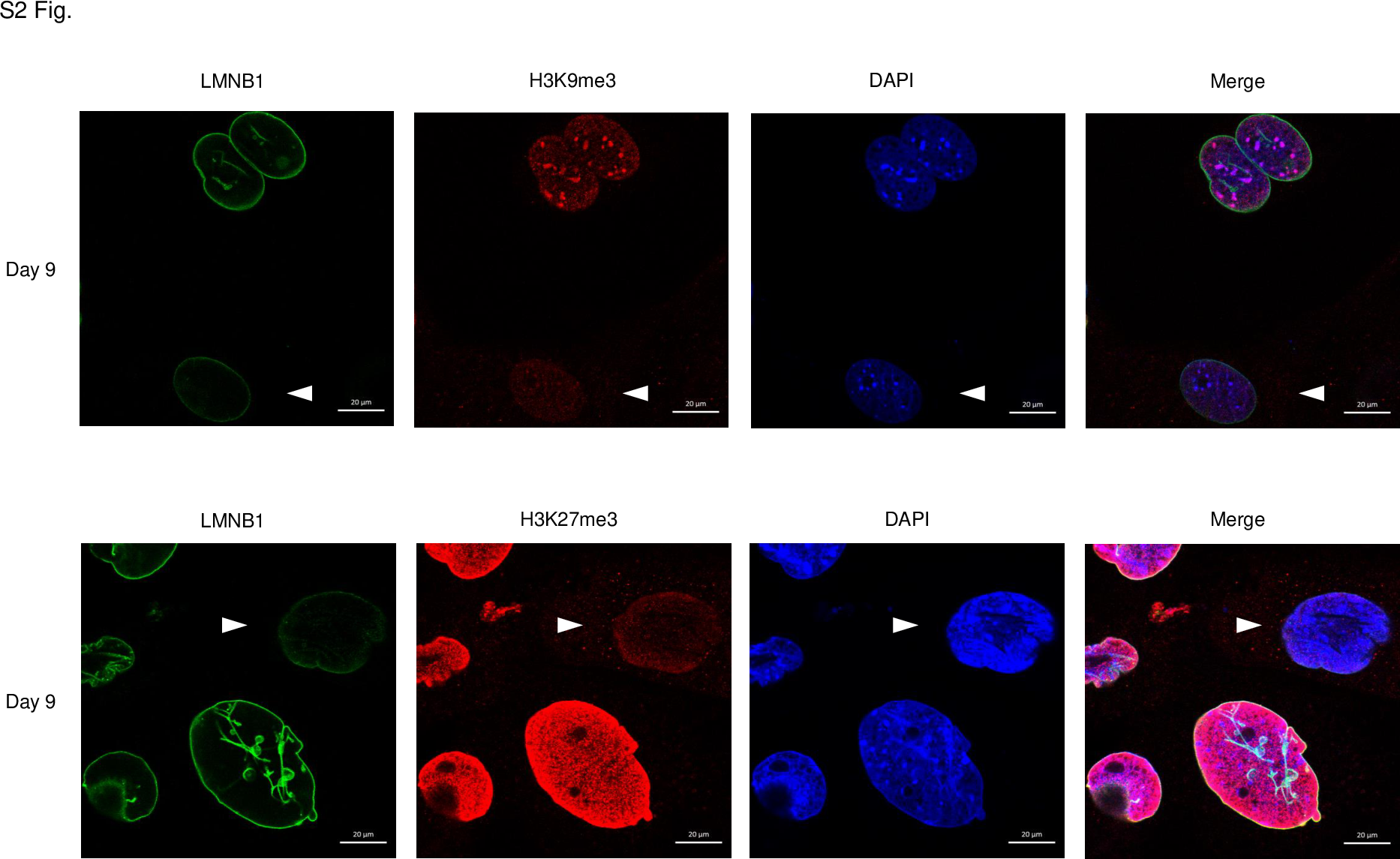

Supplement: S2 Fig — Fluorescence of H3K9me3 (top) and that of H3K27me3 (bottom) were weakened in LMNB1-loss TGCs (arrowheads). Bars mean 20 μm. (TIF) [file pone.0254674.s002.tif]

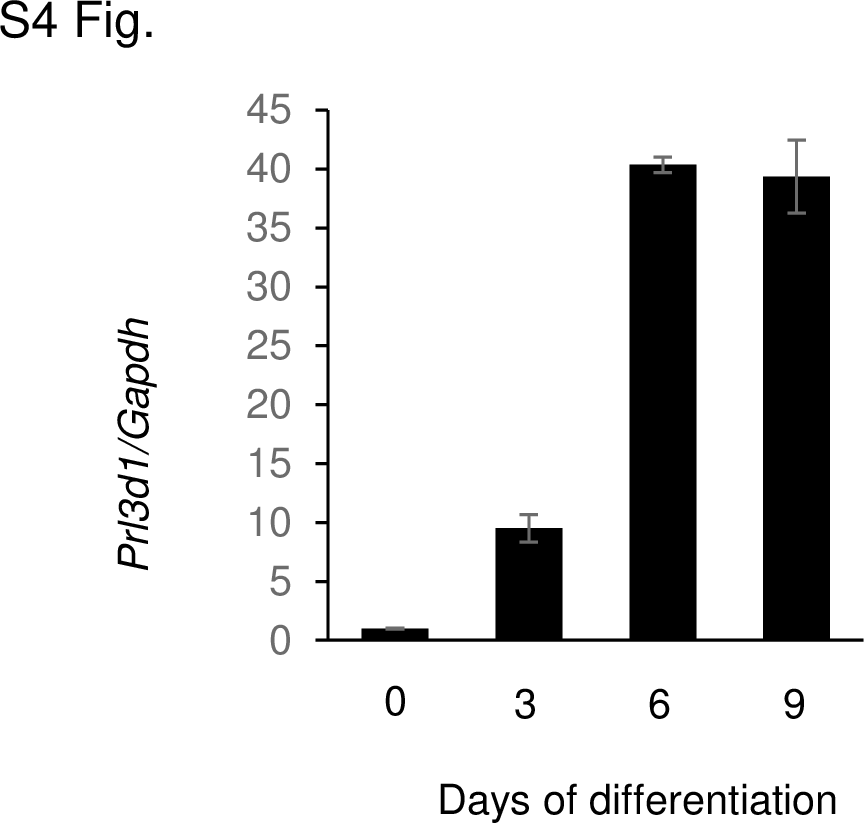

Supplement: S4 Fig — Expression levels were normalized with Gapdh. The data are presented as means ± SE (n = 3). (TIF) [file pone.0254674.s004.tif]

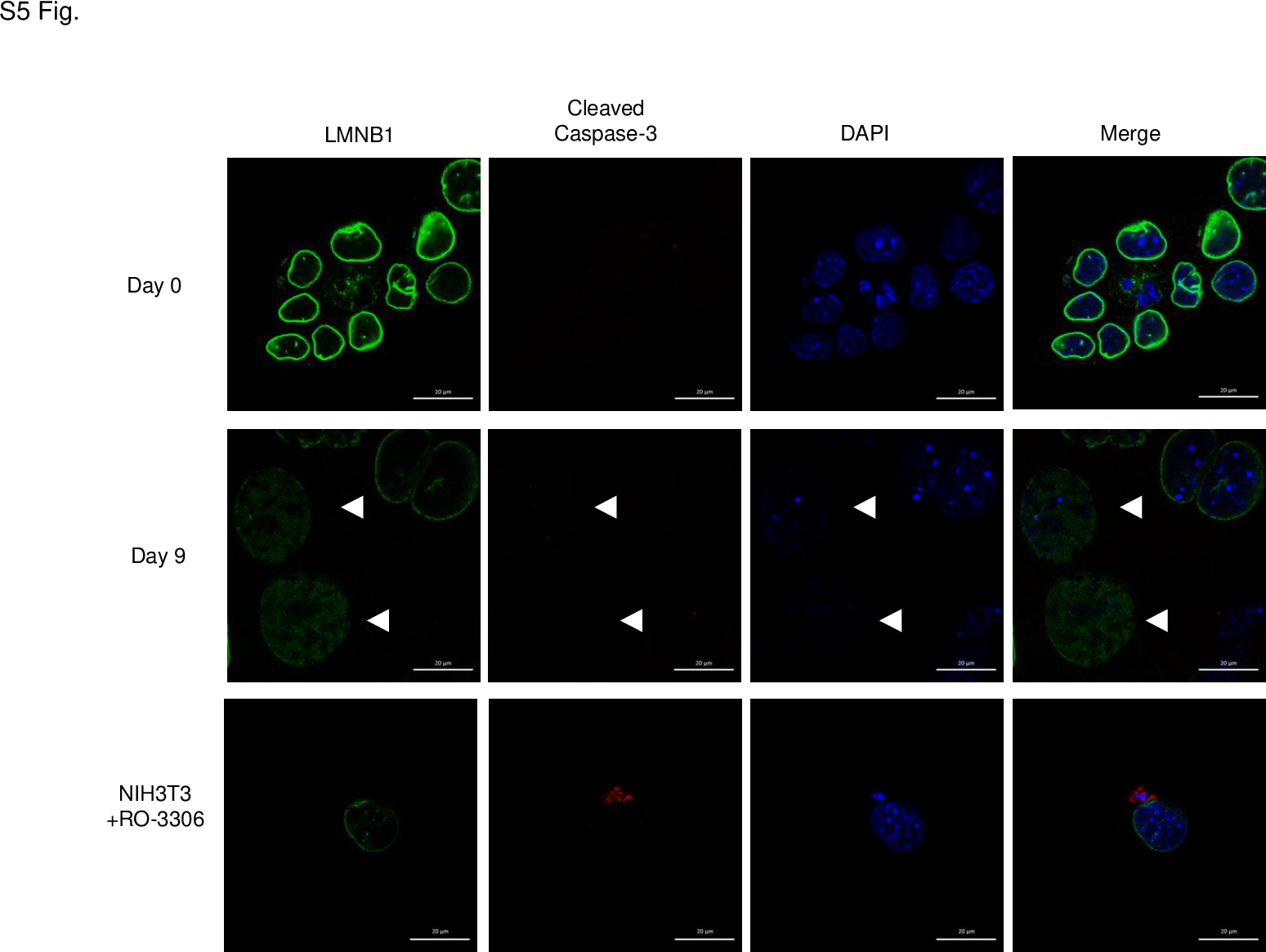

Supplement: S5 Fig — Cleaved caspase-3 was not detected in LMNB1-loss TGCs (arrowheads). To induce apoptosis, RO-3306 was added to NIH3T3 cells for 72 h at a final concentration of 10–5 M. Bars mean 20 μm. (TIF) [file pone.0254674.s005.tif]

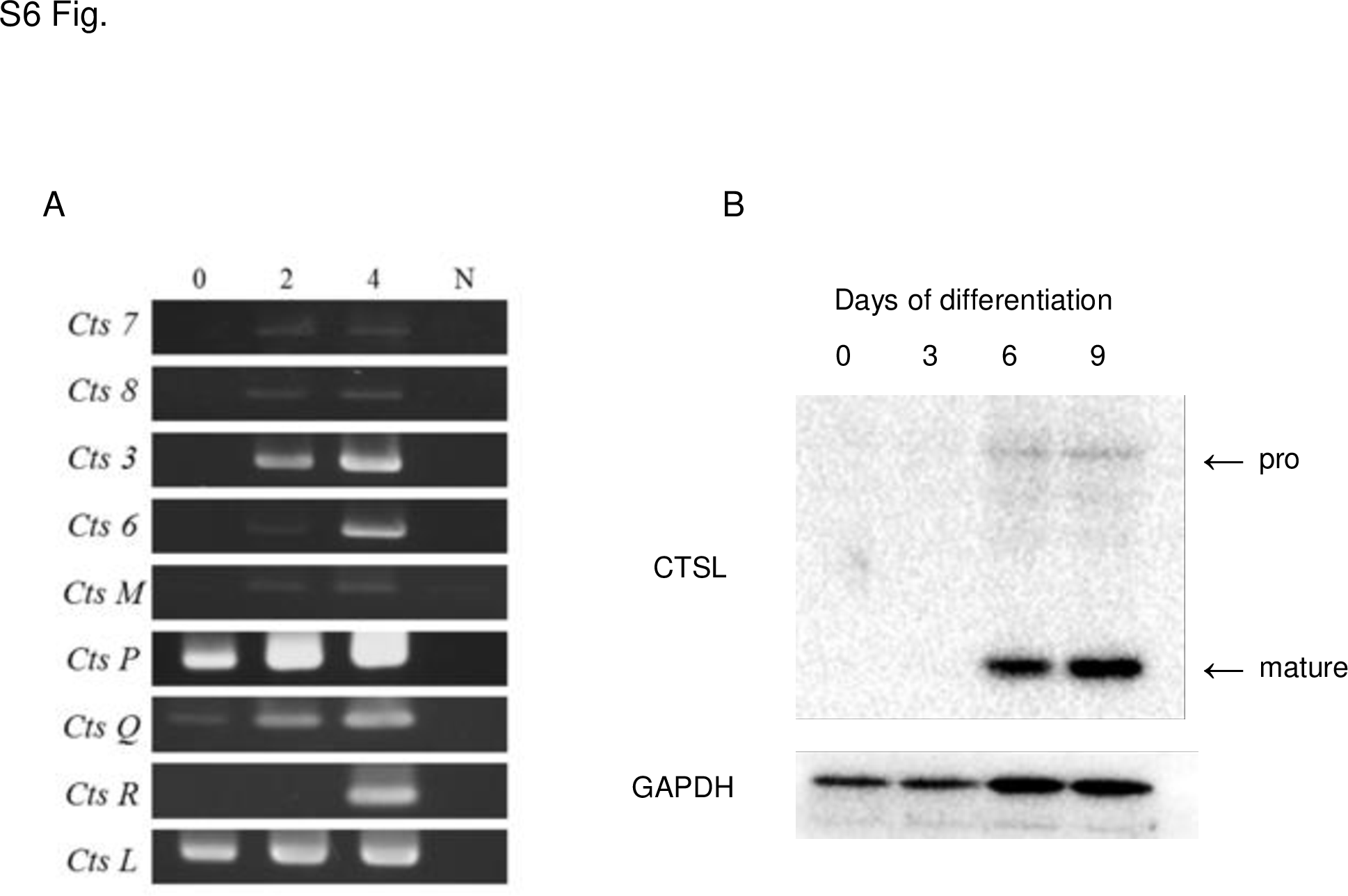

Supplement: S6 Fig — (A) RT-PCR analysis of Cts family genes. (B) Western blot analysis of CTSL protein in TS cells after differentiation. Mature CTSL increased dramatically from day 6 after differentiation. (TIF) [file pone.0254674.s006.tif]
