# Supplementary material for: Progesterone depletion results in Lamin B1 loss and induction of cell death in mouse trophoblast giant cells
Source: PLoS One. 2021 Jul 14;16(7):e0254674. doi: 10.1371/journal.pone.0254674 (PMC8279370; doi:10.1371/journal.pone.0254674)
Supplement: S1 Raw images — (PDF) [file pone.0254674.s007.pdf]

Fig3B raw images

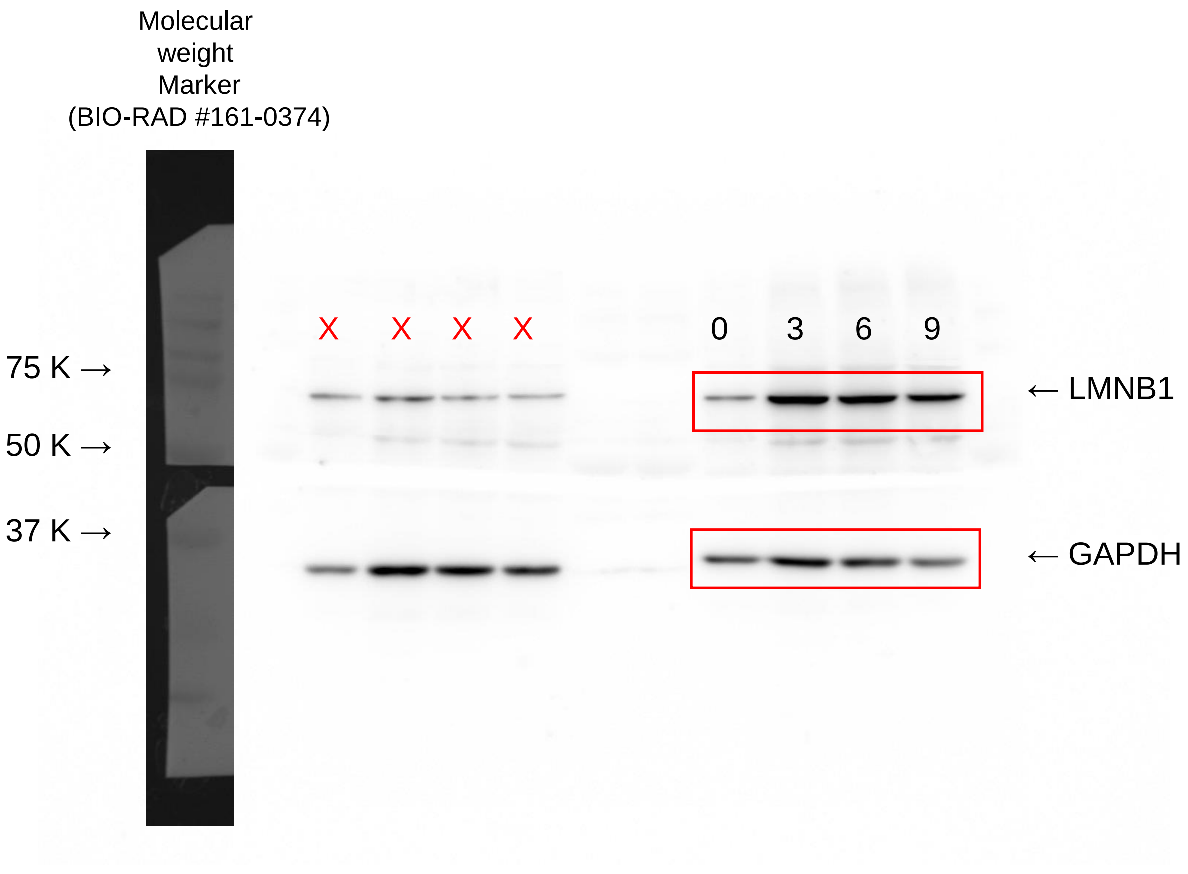

PVDF membrane was cut and images were captured at same time by LAS-3000.
Exposure time: 45sec

Fig4A raw images

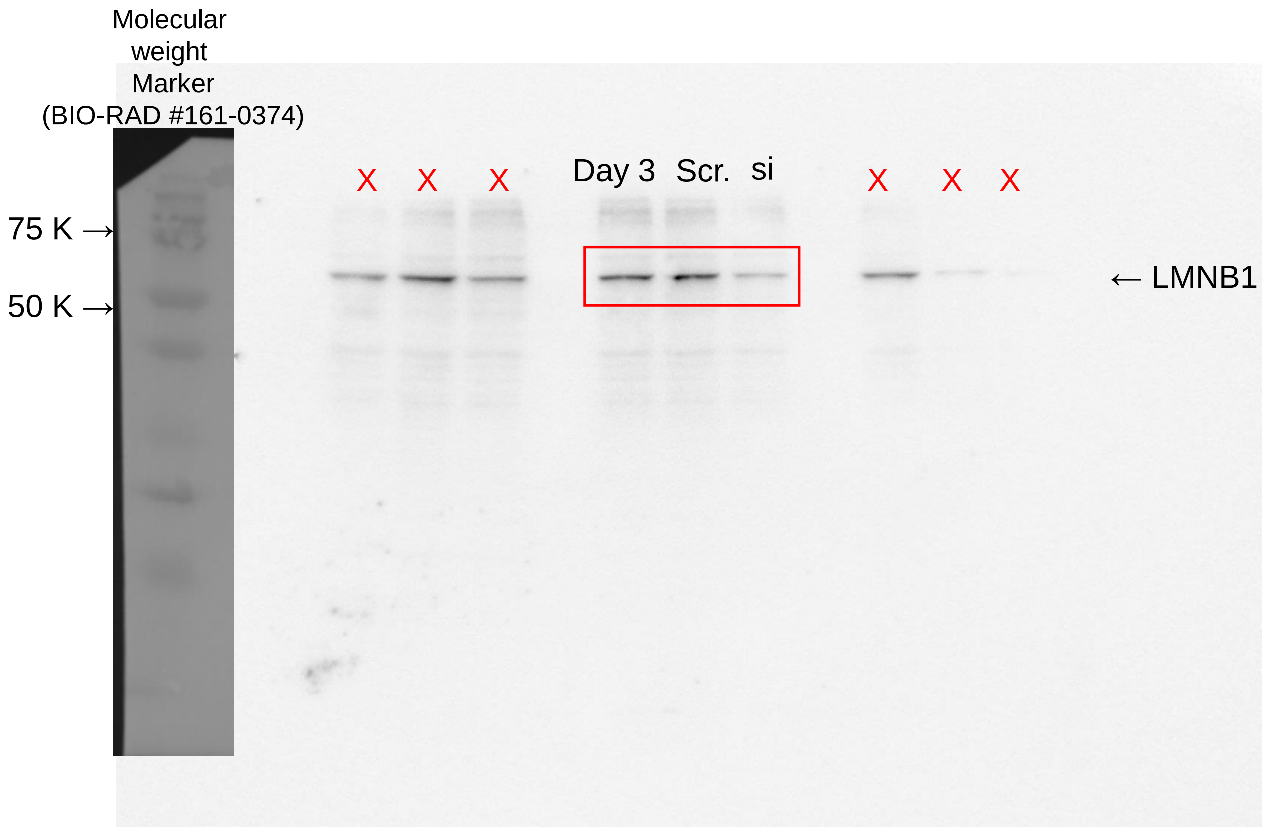

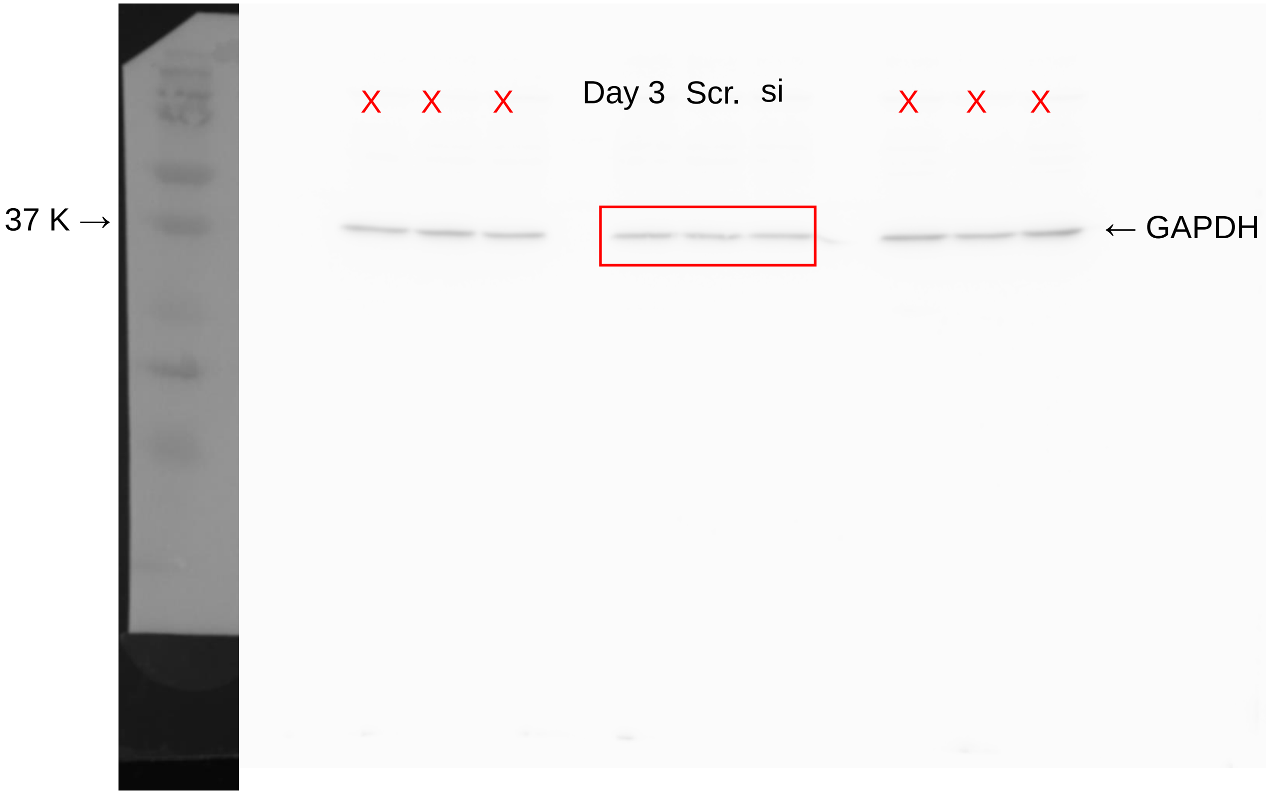

Images were captured by LAS-3000. Exposure time :45sec (LMNB1), 30sec (GAPDH)
Molecular weight marker image was captured only one time at GAPDH detection timing.
Blot images were same membrane.

S6 Fig raw images

Molecular weight Marker (#BRG-100-02)

0 2 4 NC

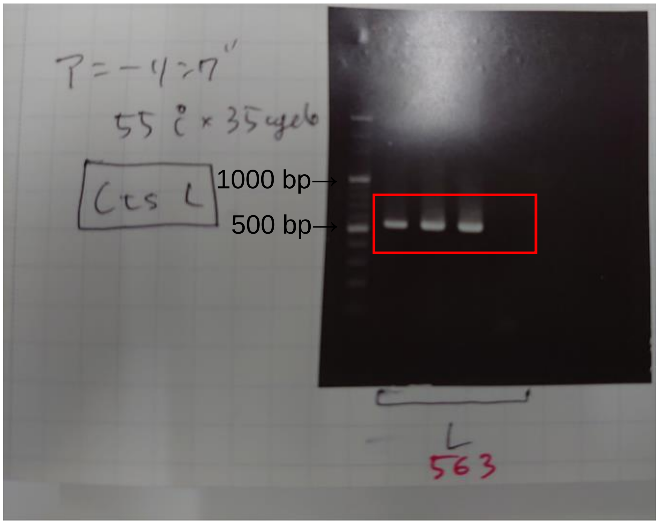

Samples were stained by ethidium bromide.
Image was captured by Gel DOC IT-TS (UVP).

Molecular weight Marker

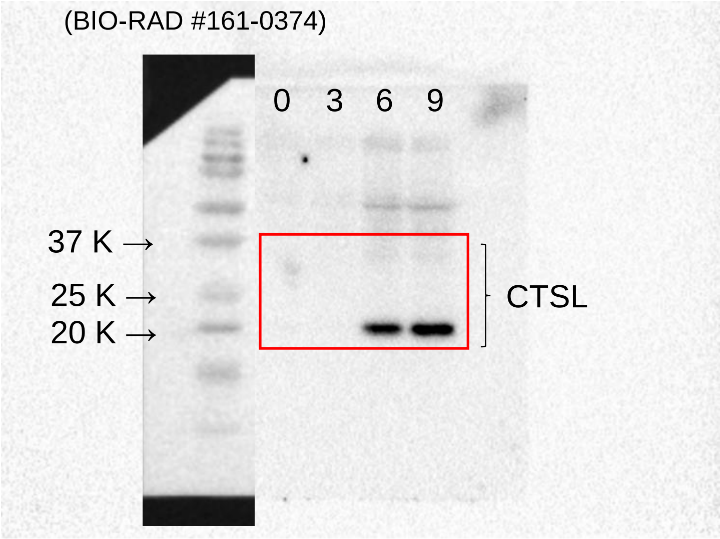

Molecular weight Marker

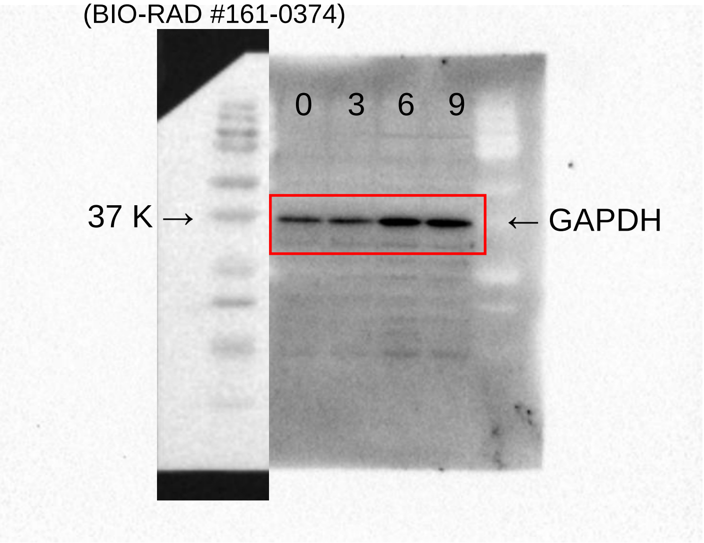

Images were captured by Chemi Doc XRS$^+$ imaging system (BIO-RAD). Exposure time : 5sec (LMNB1), 15sec (GAPDH)
Molecular weight marker image was captured only one time at CTSL detection timing.
Blot images were same membrane.
